# RETRACTION: Benign Prostatic Hyperplasia Wound after Surgical Removal in Subjects on Anticoagulant or Antiplatelet Therapy: A Meta‐Analysis

**DOI:** 10.1111/iwj.70418

**Published:** 2025-03-26

## Abstract

**RETRACTION:** JiX. , ZhaoY. , ZhangL. , and LiuY. , “Benign Prostatic Hyperplasia Wound after Surgical Removal in Subjects on Anticoagulant or Antiplatelet Therapy: A Meta‐Analysis,” International Wound Journal 19, no. 8 (2022): 1990–1999, 10.1111/iwj.13799.35419950 PMC9705179

The above article, published online on 14 April 2022, in Wiley Online Library (http://onlinelibrary.wiley.com/), has been retracted by agreement between the journal Editor in Chief, Professor Keith Harding; and John Wiley & Sons Ltd. An Expression of Concern had previously been published to alert readers to concerns regarding data presented in Tables 1 and 2 [https://doi.org/10.1111/iwj.14303]. Following an investigation by the publisher, all parties have concluded that this article was accepted solely on the basis of a compromised peer review process. The editors have therefore decided to retract the article. The authors did not respond to our notice regarding the retraction.

**RETRACTION:** X. Ji , Y. Zhao , L. Zhang , and Y. Liu , “Benign Prostatic Hyperplasia Wound after Surgical Removal in Subjects on Anticoagulant or Antiplatelet Therapy: A Meta‐Analysis,” International Wound Journal 19, no. 8 (2022): 1990–1999, 10.1111/iwj.13799.35419950 PMC9705179

The above article, published online on 14 April 2022, in Wiley Online Library (http://onlinelibrary.wiley.com/), has been retracted by agreement between the journal Editor in Chief, Professor Keith Harding; and John Wiley & Sons Ltd. An Expression of Concern had previously been published to alert readers to concerns regarding data presented in Tables 1 and 2 [https://doi.org/10.1111/iwj.14303]. Following an investigation by the publisher, all parties have concluded that this article was accepted solely on the basis of a compromised peer review process. The editors have therefore decided to retract the article. The authors did not respond to our notice regarding the retraction.

